# Paraneoplastic vasculitis associated to pelvic chondrosarcoma: a case report

**DOI:** 10.1051/sicotj/2015042

**Published:** 2016-02-26

**Authors:** Camilla Arvinius, Ana González-Pérez, Javier García-Coiradas, Roberto García-Maroto, Juan Luis Cebrián-Parra

**Affiliations:** 1 Department of Orthopaedic Surgery and Traumatology, Hospital Clínico San Carlos 28 040 Madrid Spain

**Keywords:** Chondrosarcoma, Vasculitis, Paraneoplastic, Solid tumour

## Abstract

Vasculopathic syndromes have been associated with hematological and solid organ malignancies. The pathogenesis of these syndromes remains largely unknown and there are no biologic markers identified. Whether it is or is not a paraneoplastic syndrome is under discussion, the close temporal relationship of cancer and vasculitis suggests that these vasculitides are a paraneoplastic condition. We report a case of a 45-year-old female patient with pelvic chondrosarcoma who underwent surgical treatment and started to present visual loss, systemic inflammatory response syndrome (SRIS), cardiac insufficiency, hepatosplenomegaly, cholestasis as well as pulmonary bleeding suggesting a sarcoma-associated vasculitis. All antibodies were negative as in secondary vasculitis. After corticoideal therapy the vasculitis resolved and at 3-year follow-up the patient had not showed any further medical complications or recurrences of the vasculitis. The parallel evolution of the vasculitis and the solid tumor combined with the resolution of the vasculitis after corticotherapy enhances the likelihood of a paraneoplastic vasculitis associated with a chondrosarcoma according to literature review.

## Introduction

Generally, neoplastic disorders can be associated with a large number of vasculopathic syndromes that affect both the venous and arterial vascular trees [1]. However, the coexistence of vasculitis and malignancy is rare, and paraneoplastic vasculitides represent less than 5% of all the vasculitides. To be considered paraneoplastic the vasculitis has to be concurrent with the primary malignancy (commonly within 12 months) and an effective treatment of the malignancy associates a resolution of the vasculitis while a recurrence of the vasculitis is generally associated with a progression of the malignancy [1–4]. The pathogenesis of this syndrome is unknown and it has been reported to occur prior to discovery of the neoplasm, concurrently with it or after malignancy recognition [1, 2]. In most patients the neoplastic disease is a hematological malignancy, making the coexistence of vasculitides and solid tumors a rare pathology. The most frequent phenotypes of vasculitis are the cutaneous vasculitis and polyarteritis nodosa. Patients with antineutrophil cytoplasmic antibodies (ANCA)-associated vasculitides present a higher risk to have an underlying primary tumor than those with Henoch-Schönlein purpura (HSP) and the patients with malignancies are generally older and more likely to be male than those without malignancies [1]. We present a case of a patient with pelvic chondrosarcoma who underwent surgical treatment and presented an associated vasculitis [2]. Since it was diagnosed during the immediate postoperative period and evolved favorably without any recurrences of either the primary tumor or the vasculitis, it was diagnosed as a paraneoplastic vasculitis. So far, there are no cases to be found that correlate a paraneoplastic vasculitis with a bone tumor, but as described when associated to other solid tumors, it resolved without leaving any sequelae after corticosteroid treatment.

## Case

A 45-year-old woman, without any prior medical history of interest, was referred with a two month history of inguinal mechanical pain with radiation to the anterior zone of the right leg and a weight loss of 2 kg. The first physical exam described inguinal pain without detecting any clinical significant abnormalities.

**Figure 1.**
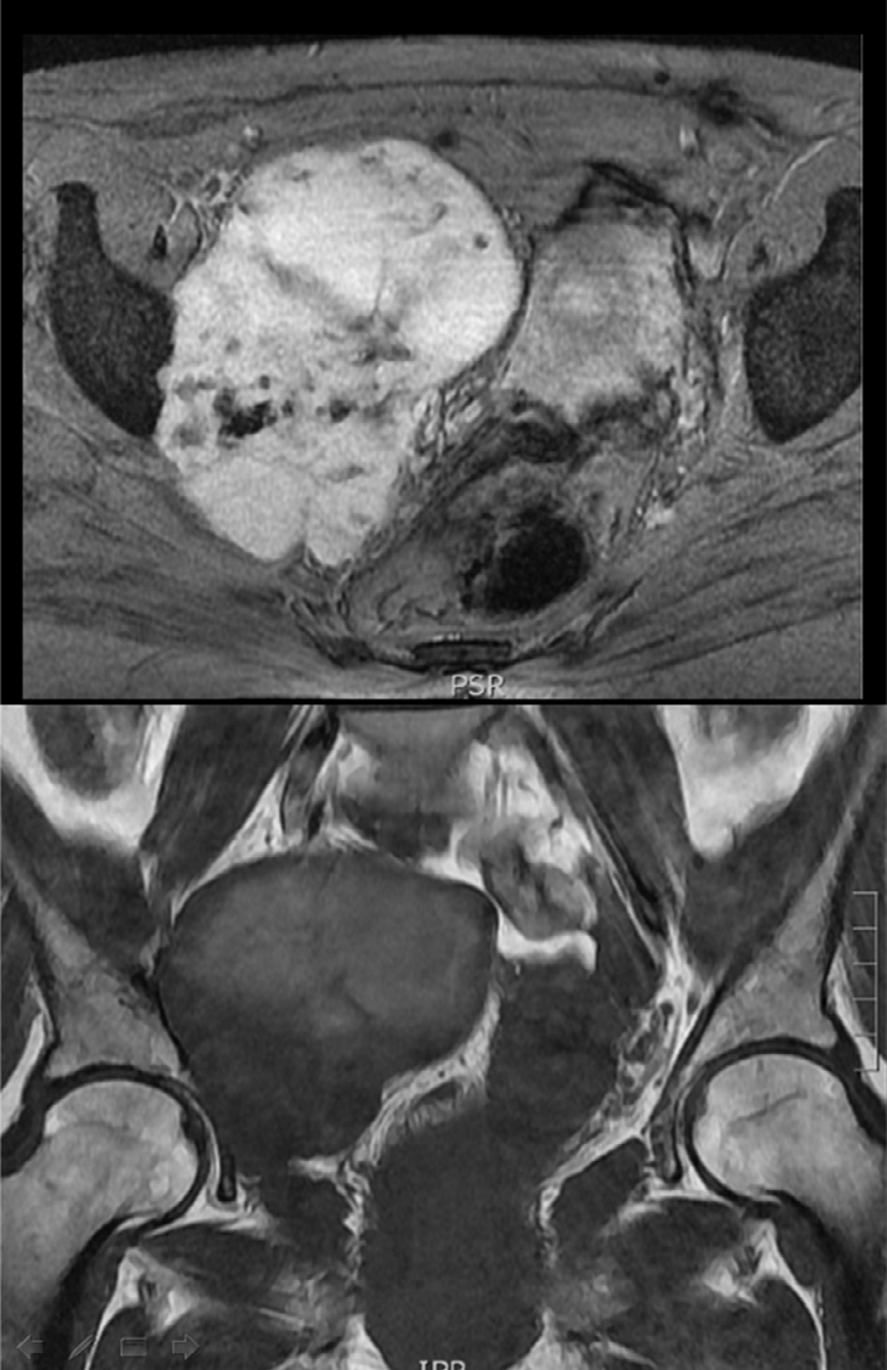
Magnetic resonance imaging (MRI) showing a polylobulated mass of soft tissue (11.2 × 7.6 × 9.1 cm) in the right obturator foramen affecting the internal obturator muscle with erosion of the acetabulum.

A magnetic resonance imaging (MRI) study was performed detecting a polylobulated mass of soft tissue and a CT-scan showed a a non-specific mass (Figure 1). The result of anatomopathological study was a chondroid tumor, suggesting a low-grade chondrosarcoma. The extension study did not show any invasion.

As to the surgical treatment, an ilioinguinal incision associated with a Watson-Jones antero-lateral approach to the hip. The tumoral mass and the complete zone II of Enneking were resected preserving the vascularization of the mayor gluteal muscle. Arthroplasty was performed with an ice-cream cone reconstruction of the pelvis (Coned, Stanmore^®^).

During the immediate postoperative care the patient showed a decreased visual acuity and color vision loss receiving an urgent evaluation by an ophthalmologist and a neurologist suspecting an ischemic etiology.

A few days later the patient presented with dyspnea, fever of 38 °C and the oxygen saturation was 84%. A CT-scan was compatible with respiratory distress possibly due to an SRIS, cardiac insufficiency, hepatosplenomegaly, and cholestasis. A bronchoscopy detected pulmonary bleeding. With the suspicion of a sarcoma-associated leukocytoclastic vasculitis an immunological study was performed with negative ANCA. Treatment with a bolus of 500 mg of methylprednisolone was given after which fever, pulmonary infiltrates, hepatosplenomegaly, and cholestasis remitted. After further oral immunosuppressive corticosteroid therapy, the inflammatory parameters were suppressed and a gradual remission of the visual disturbance was obtained.

**Figure 2.**
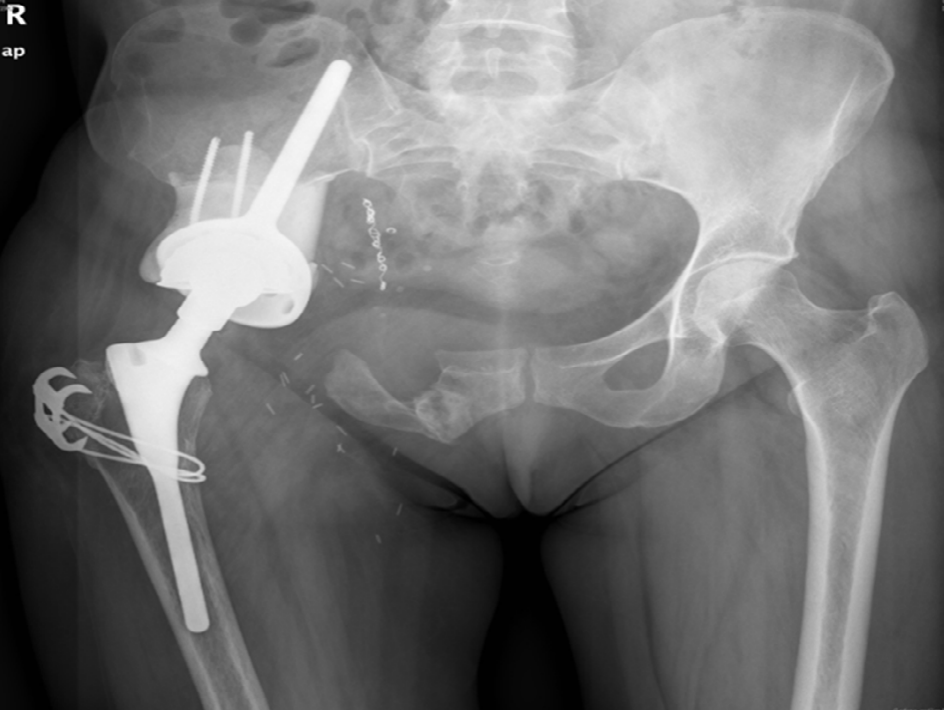
Radiographic result at 3-year follow-up.

The anatomopathology showed a well-differentiated chondrosarcoma (grade I) of 11 × 8 × 7 cm without involvement of the surgical borders. At 6-month follow-up a thoracoabdominal CT-scan showed a complete remission. At the 3-year follow-up the patient uses a cane for stability and has an orthosis to correct a 2 cm leg length discrepancy. She performs activities of daily life independently and no further medical complications or recurrences of the vasculitis have been observed (Figure 2).

## Discussion

The coexistence of vasculitis and solid tumors, as seems to be our case, is a rare disorder [2, 3, 5]. Seventy seven percent of patients who present a tumor-vasculitis association have a hematological malignancy while only 17% presents with a solid tumor [2]. The para-neoplastic vasculitides may be of small, medium, and large-sized vessels, but small vessel vasculitis is the most frequently observed [1]. In decreasing order, the most common solid malignancies described in association with vasculitis are lung, colon adenocarcinoma, renal carcinoma, cancer of the urinary bladder, and prostate and breast cancer. The types of vasculitides more often associated with a neoplasia are leukocytoclastic vasculitis, polyarteritis nodosa, Henoch-Schönlein purpura, and polyarteritis nodosa [1, 2].

Like many other para-neoplastic syndromes, the pathogenesis remains largely unknown and there are no biologic markers identified [1–3]. Nevertheless, some theories have been proposed to explain the mechanism of the vascular injury:

1. the invasion of cancer cells in blood vessel walls triggers an immunologic response against these;
2. a release of substances such as enzymes or chemotactic factors might damage the vascular endothelium;
3. the cancer cells act as sensitizing agents capable of eliciting a reaction against the host;
4. the tumor releases antigens that cause antigen-antibody complexes whose deposition on the blood vessel wall causes an inflammatory response;
5. the production of a thrombus or embolus might destroy the vessel walls via a direct mechanical effect [2].

Whether it is or not a para-neoplastic syndrome is under discussion. To be considered para-neoplastic, its course has to be parallel to the underlying tumor and reappear in case of recurrence [4]. The temporal relationship between both conditions suggests a para-neoplastic origin; its appearance varies from 25 months proceeding to nine months following cancer diagnosis [2]. There also seems to be a concordance of disease activity and treatment response for both cancer and vasculitis [1, 3]. The clinical manifestations of para-neoplastic vasculitides are similar to those seen in patients with primary vasculitis [4]. The laboratory findings are generally nonspecific and include normocytic normochromic anemia, increased erythrocyte sedimentation rate, and elevated fibrinogen levels or proteinuria in patients who develop HSP [1]. As to antibodies, antinuclear antibodies (ANA) can be detected in up to 53% of the patients, rheumatoid factor (RF) in 20% cases, and ANCA were negative in all cases. The latter may be present in the blood in primary systemic vasculitis, but are usually negative in secondary vasculitis as in our case [2].

As to the treatment, glucocorticoid therapy possibly associated with immunosuppressive agents is generally used to treat the vasculitis. However, since it is a para-neoplastic syndrome, the best results are obtained in combination with surgical removal of the primary cancer. In most cases, radical cancer treatment is followed by resolution of the vasculitis and its recurrences usually occur with tumor recurrences, enhancing the vasculitis as a para-neoplastic syndrome [1]. Although the vasculitis has been described as presenting a parallel evolution to the cancer, an independent evolution has also been described in previous reports [2]. Solans-Laqué et al. have described some recommended guidelines for diagnosing a vasculitis as a para-neoplastic process: temporal relationship; consistency of the relationship between the evolution of both pathologies; and an unexpected frequency between the two conditions.

As to our case, even though the surgery is not exempt from complications, there are, at this moment, no valid treatment options than surgery for this kind of tumor. In our experience, the reconstructive surgery permits acceptable functional outcomes in these patients even though the medical complications should also be taken into consideration. Our patient presented a vasculitis within the first month after the resection of a chondrosarcoma without any relevant medical history. The immunologic study did not show any significant alteration and the negative ANCA suggests a secondary vasculitis. Furthermore, the parallel evolution of the vasculitis and the solid tumor combined with the resolution of the vasculitis after corticosteroidtherapy enhances the likelihood of a para-neoplastic vasculitis associated with a chondrosarcoma according to the literature review.

## Conflict of interest

All authors state that they have no conflicts of interest.
